# The Use of Copper Oxides as Cross-Linking Substances for Chloroprene Rubber and Study of the Vulcanizates Properties. Part II. The Effect of Filler Type on the Properties of CR Products

**DOI:** 10.3390/ma14216528

**Published:** 2021-10-29

**Authors:** Aleksandra Smejda-Krzewicka, Piotr Kobędza, Krzysztof Strzelec, Agnieszka Adamus-Włodarczyk

**Affiliations:** 1 Faculty of Chemistry, Institute of Polymer and Dye Technology, Lodz University of Technology, Stefanowskiego 16, 90-537 Lodz, Poland; piotr.kobedza@dokt.p.lodz.pl (P.K.); krzysztof.strzelec@p.lodz.pl (K.S.); 2 Faculty of Chemistry, Institute of Applied Radiation Chemistry, Lodz University of Technology, Wroblewskiego 15, 93-590 Lodz, Poland; agnieszka.adamus@p.lodz.pl

**Keywords:** copper oxides, chloroprene rubber, cross-linking, filler, mechanical properties, morphology, Payne effect, Mullins effect, flammability

## Abstract

The properties of rubber materials are dependent on the characteristics of the elastomer matrix, the filler type, the cross-linking agent, the number of ingredients, and their interactions. In the previous article, we showed that chloroprene rubber can be efficiently cross-linked with copper(I) oxide or copper(II) oxide. During the processing of rubber compounds, the incorporation of a filler and a curing substance are two substantial parameters, such as the homogeneity of mixing and cross-linking that significantly affect the properties of the vulcanizates. Therefore, this work aimed to evaluate the curing characteristics, mechanical and dynamical properties, morphology, and flammability of the composites containing chloroprene rubber cross-linked with Cu_2_O or CuO and filled with different fillers (silica, carbon black, montmorillonite, kaolin, chalk). It was found that the type of filler and curing agent had a significant impact on the degree of cross-linking of the chloroprene rubber and the properties of its vulcanizates. The degree and speed of the cross-linking of filled CR were higher when the CR was cured with copper(II) oxide. Among the fillers used, the presence of carbon black or silica ensured the highest degree of CR cross-linking and the most useful properties. The flammability tests indicated that all produced vulcanizates were characterized by a high oxygen index, which allows them to be classified as non-flammable materials.

## 1. Introduction

Currently, commercially available rubber products are usually filled materials. Fillers are comminuted inorganic or organic substances that form a dispersed, insoluble phase in the rubber mixture and are used to produce rubber products with the desired properties. Homogeneous filler distribution and dispersion within the elastomer matrix are also dependent on the fillers (van der Waals interactions and geometry) [1,2,3,4], polymer type [3,5], and on processing method [4]. It is important to disperse fillers properly in the polymer matrix to avoid the formation of their agglomerates. An important function of the fillers is to facilitate the processing of the mixture (especially during extrusion or calendering). Thus, fillers are solid materials capable of changing the physical and chemical properties of elastomers by surface interactions or their lack thereof and by own characteristics [6]. The reinforcement effect by the incorporation of a select filler into the rubber matrix can be associated with strong physical or chemical interactions between the filler particles and the elastomer phase. The filler–elastomer interactions include chemisorption or physical adsorption. The specific surface area and the concentration of functional groups present on the filler surface are very important factors in determining the final properties of the cross-linked elastomer [7,8,9].

Due to the influence on the properties of vulcanizates, three classes of fillers are generally recognized: active (reinforcing), semi-active (semi-reinforcing), and inactive (non-reinforcing) [10,11,12]. These definitions are vague as the effects of the selected filler may depend on the type of elastomer. However, generally active fillers significantly improve the properties of vulcanizates (e.g., mechanical strength, abrasion resistance). These include, among others, activated carbon black, silica, and aluminosilicates. Semi-reactive fillers (e.g., kaolin, magnesium carbonate) increase the tensile strength indirectly but not the abrasion resistance. On the other hand, non-reinforcing fillers do not improve the mechanical properties of vulcanizates, but significantly reduce the cost of production. Chalk, talc, barium sulfate, and diatomaceous earth are inactive fillers. 

The effect of achievable reinforcement is largely determined by the particle size, assuming an appropriate level of adhesion of particles to the selected rubber. Generally, the active fillers have primary particle sizes of less than 100 nm, with semi-active filler particles in the range from 100–500 nm, while the inactive fillers have particle sizes above 500 nm. There is also another class of fillers called diluents, their sizes exceed 5000 nm, and these fillers significantly degrade the properties of rubber [12]. The improvement in vulcanizate properties through the addition of a filler is dependent not only on the filler size but also on the shape of the filler, its surface characteristics, and the dispersion of the filler particles in the elastomer matrix [13,14,15]. The evaluation of the reinforcing effect of fillers is usually based on the determination of the processability of the rubbers, and the mechanical and dynamic properties of the cured materials [16,17]. 

In silica applications, the subclass of tektosilicates is of greatest interest [6]. Four minerals (quartz, tridymite, cristobalite, and opal) belong to the silica group and three of them (quartz, cristobalite, and opal) are used as fillers or materials for their production. In addition to natural products, synthetic materials are commonly used. Two methods of production are used, the pyrogenic or thermal (known as fumed silica) and wet process (known as precipitated silica). Precipitated silica (for example, silica named Arsil) is produced from sodium silicate by its reaction with sulfuric and hydrochloric acids. Precipitated silica is used often because its satisfactory dispersion limits the formation of its agglomerates. Many articles have revealed that precipitated silica could interact strongly via primary chemical bonds with functionalized elastomers such as epoxidized natural rubber, carboxylated butadiene–acrylonitrile rubber, chlorosulfonated polyethylene, or chloroprene rubber [18,19,20,21]. Silica has been used as a reinforcing filler in elastomer technology for a long time, and, thus, reinforced rubber products are suitable materials for industrial practice [17,22]. Silica improves tear strength, abrasion resistance, and reduces rolling resistance in tires [23,24]. 

Carbon black is another commonly used reinforcing filler. It is composed of large sheets of hexagonal rings formed by carbon atoms separated from each other by a distance of 0.142 nm [6]. The layers of carbon black are parallel to each other but not arranged in order, usually forming concentric inner layers. Such an arrangement is called a turbostratic structure. Carbon black is a filler that affects the physico-mechanical properties of the vulcanizate, such as the degree of swelling, water absorption, tensile strength, elongation at break, and tinting strength. As the specific surface of carbon black increases, the tensile and tear strength, as well as the hardness and abrasion resistance of the vulcanizates increase, while the elongation at the break decreases [25]. However, the morphology of carbon black and its tendency to form agglomerates make the manufacturing process of such filled materials very difficult. 

Active fillers also include nanofillers, e.g., montmorillonite (MMT), which belongs to layered silicate clays. It is a hydrophilic nanofiller with the chemical composition of Al_2_O_3_·4SiO_2_·3H_2_O. This filler has two layers of a tetrahedron of Si–O and one layer of an octahedron with Al–O in between [26]. To achieve the desired performance and increase interaction, the clay aggregates must be exfoliated in the elastomer matrix [27]. When MMT is incorporated into the polymer matrix, three types of structures are commonly obtained. These structures are a phase-separated structure, an intercalated structure, and an exfoliated or delaminated structure. An exfoliated structure is always expected to form a true nanocomposite because of the superior interfacial adhesion.

An example of a semi-reinforcing filler is kaolin, produced by the decomposition of granite and white feldspar [6]. Kaolin is one of the most used fillers in the rubber industry. Kaolin, like silica, can adsorb basic chemicals, which can slow vulcanization. Kaolin is ground (dry or wet) and calcined then wet cleaned and calcined between 400 and 600 °C [25]. Before applying kaolin, grinding it properly is crucial. The grinding process reduces the size and delaminates the stacks resulting in a finer product. The chemical formula of kaolin is Al_2_O_3_·2SiO_2_·2H_2_O. The particles of this filler are composed of stacks that can form aggregates closer together in a spherical shape. 

Chalk is classified as an inactive filler. Chalk, as a filler, is ground calcium carbonate of natural or synthetic origin, usually contaminated with small amounts of iron compounds, aluminum, silicon, or magnesium. Modified chalk that has been precipitated in some polar rubbers may exhibit reinforcing properties [25]. The presence of chalk in elastomers facilitates the calendering and extrusion of rubber mixtures and obtaining a smooth surface of semi-finished and finished products. Chalk-filled mixtures fill the vulcanization forms very well.

Most of the commercially available rubber products are filled materials, thus, in this work, we have decided to investigate how the classically used fillers will affect the properties of the new elastomer compounds designed by us. The choice of chloroprene rubber (CR) was chosen because of its unique features. CR is a multipurpose synthetic elastomer with a broad industrial application (including wire and cable jacketing, hose, tubes, covers, automotive gaskets, seals, cellular products, construction applications, asphalt modification) due to its high ozone resistance, extraordinary oil and fuel resistance, toughness, good adhesion to other materials, and high heat resistance [6,28,29]. 

This study aimed to prepare chloroprene rubber composites filled with different fillers. Additionally, detailed investigations on the role of copper(I) oxide and copper(II) oxide as curing agents in CR compounds were thoroughly investigated. The effects of copper oxides on cross-linking and CR properties were described in detail in our earlier article [30]. This study will lead to a scientific understanding of how the type of copper oxide influences the properties of filled CR vulcanizates and will extend to being a source of useful information for preparing rubber products based on CR. In this paper, the roles of silica, carbon black, montmorillonite, kaolin, and chalk on the curing characteristics, mechanical and dynamical properties, and flammability of CR composites were investigated. 

## 2. Experimental Part

### 2.1. Materials

In this study, chloroprene rubber, CR (Baypren^®^216 MV from Lanxess GmbH, Cologne, Germany), with a density of 1.23 g/cm^3^ and Mooney viscosity (ML 1 + 4 100 °C) of 43 ± 5 was used. As cross-linking agents, two copper oxides were used: copper(I) oxide, Cu_2_O (POCH S.A., Gliwice, Poland), with a density of 6.00 g/cm^3^, pureness of >99% and particle size of ≤7 µm, and copper(II) oxide, CuO (Sigma-Aldrich Chemie GmbH, Steinheim am Albuch, Germany), with a density of 6.32 g/cm^3^, pureness of 98% and particle size of <10 µm. Stearic acid, SA (Chemical Worldwide Business Sp. z o. o., Słupca, Poland) with a density of 0.85 g/cm^3^ was used as a dispersing agent.

The following fillers of elastomer composites were used:

- precipitated silica Arsil (Z. Ch. Rudniki S.A., Rudniki, Poland) with a bulk density of ~150 g/dm^3^ and pureness of >95%;
- technical chalk (POCH S.A., Gliwice, Poland) with a density of 2.71 g/cm^3^ and grain size of <10 µm (99.7%);
- technical kaolin (POCH S.A., Gliwice, Poland) with a density of 2.60 g/cm^3^ and an average grain size of 1,3 µm;
- nanofiller NanoBent, MMT (ZGM “Zębiec”, Zębiec, Poland), montmorillonite modified by ammonium salt-containing hydroxyl groups, with an average grain size from 20–60 µm (81%), ≤20 µm (19%), and a layer spacing from 3.8–3.9 nm;
- carbon black, CB (Chemical Worldwide Business Sp. z o. o., Słupca, Poland), with a bulk destiny of 380 g/dm^3^ and a surface area of 78 m^2^/g.

### 2.2. Research Methods

Chloroprene rubber composites were prepared using a Krupp-Gruson laboratory two-roll mill (Laborwalzwerk 200x450, Krupp-Gruson, Magdeburg-Buckau, Germany) with a roll diameter of 200 mm and a length of 450 mm. The temperature of the roll was between 20 and 25 °C, while the speed of the front roll was 200 rpm, with roll friction of 1:1.25. The constituents were added in the sequence presented in Table 1. First, the rubbers were plasticized, then the stearic acid and the cross-linking agent (Cu_2_O or CuO) were incorporated. Finally, the filler (silica, chalk, kaolin, MMT, or carbon black) was added. The preparation of one composite lasted 10 min and then it was conditioned for 24 h.

Vulcametric measurements were determined by the Alpha Technologies MDR 2000 rotorless rheometer, heated to 160 °C. The oscillation frequency was 1.67 Hz. The test lasted 60 min and was performed according to the ASTM D5289-17 standard. The test consisted of registering the torque (*M*) as a function of time (*t*) during the cross-linking of the test sample at a constant temperature (*T*). The torque value depends on the stiffness of the rubber mixture and changes as the cross-linking process progress. Based on the vulcametric curves (*M* = f (*t*)), the optimal cross-linking time (*t*_90_—the time at which the torque reaches 90% of the increase), the scorch time (*t*_02_), the minimum torque (*M*_min_), and the torque increment after a specified heating time (Δ*M_t_*) were determined. The cure rate index (*CRI*), a measure of the cross-linking rate, was calculated from Formula (1):

(1) $$CRI=\frac{100}{t_{90}-t_{02}}$$

The prepared samples were then vulcanized using an electrically heated hydraulic press. The vulcanization parameters were as follows: temperature, 160 °C; pressure, 150 bars; time, 45 min. 

The determination of equilibrium swelling was performed. Samples were cut from prepared vulcanizates in four different shapes. Each of them weighed from 25 to 50 mg, with an accuracy of 0.1 mg. The samples were then placed with the solvents toluene or heptane, in weighing vessels. The prepared samples were placed in a thermostatic chamber for 72 h at 25 °C, which was then bathed with diethyl ether, dried on filter paper, and then weighed again. The samples were then dried in a dryer at a temperature of 50 °C to a constant weight and they were reweighed. The equilibrium volume swelling degree in toluene or heptane (*Q_v_*) was calculated from Formula (2):

(2) $$Q_{v}=Q_{w}\times \frac{d_{v}}{d_{s}}$$

where *Q_w_* is the value of the equilibrium mass swelling (mg/mg), *d*_v_ is the vulcanizate density (g/cm^3^), and *d*_s_ is the solvent density (g/cm^3^). 

The equilibrium weight swelling was calculated from Formula (3):

(3) $$Q_{w}=\frac{m_{s}-m_{d}}{m_{d}^{*}}$$

where *m*_s_ is the swollen sample weight (mg), *m*_d_ is the dry sample weight (mg), and m*_d_ is the reduced sample weight calculated from Formula (4):

(4) $$m_{d}^{*}=m_{d}-m_{0}\times \frac{m_{m}}{m_{t}}$$

where *m*_0_ is the initial sample weight (mg), *m*_m_ is the mineral substance content in the compound (mg), and *m*_t_ is the total weight of the compound (mg).

The degree of cross-linking (α_c_) was determined from Formula (5):

(5) $$\alpha_{c}=\frac{1}{Q_{v}}$$

Tensile properties were measured according to the PN-ISO 37: 2007 standard using a ZwickRoell machine (model 1435) connected with the appropriate computer software. In the study, samples in the shape of B-type paddles with a measuring section width of 4 mm were used. The scope of the property tests was included: stress at 100%, 200%, and 300% of elongation (*S*_e100_, *S*_e200_, *S*_e300_), tensile strength (*TS*_b_), and elongation at the break (*E*_b_).

The Mullins effect (*M*_E_) was calculated from the hysteresis loop measurements according to Formula (6):

(6) $$M_{E}=\frac{W_{1}-W_{5}}{W_{5}}\times 100\%$$

where *W*_1_ is the hysteresis loss at the first stretching of the sample [kJ/m^2^] and *W*_5_ is the hysteresis loss at the fifth stretching of the sample [kJ/m^2^].

Measurements of the Payne effect (∆*G*′), storage modulus (*G*′), and loss modulus (*G″*) were made on the vulcanizate discs with a diameter of 25 mm and a thickness of 2 mm using TA Instruments’ ARES-G2 rotational rheometer (New Castle, UK) according to the ISO 4664:2011 standard. The parameters used were as follows: a soak time of 10 s, an angular frequency of 10 rad/s, a logarithmic sweep with strain from 0.005 to 70% s, 20 points per decade, and an initial force of 5 N. The experiment was carried out at room temperature. The modulus of elasticity (G′) describes the elastic properties of the vulcanizate and is proportional to the energy retained during its deformation. The loss modulus (G″) describes the viscous properties and is proportional to the amount of work converted into heat during the deformation. The storage and loss moduli values enabled the calculation of the Payne effect according to Formula (7):

(7) $$∆G^{\prime }={G^{\prime }}_{\max}-{G^{\prime }}_{\min}$$

where *G′*_max_ is the maximum value of the storage modulus (MPa), and *G′*_min_ is the minimum value of the storage modulus (MPa).

The surface morphology of the vulcanizates was evaluated using a scanning electron microscope (SEM) Hitachi Tabletop Microscope TM-1000 (Tokyo, Japan). The preparation of samples for measurement consisted of placing a double-sided self-adhesive foil on special tables and gluing the tested sample to it. Then, a gold layer was applied to the prepared sample using the Cressington Sputter coater 108 auto vacuum sputtering machine (Redding, CA, USA) at a pressure greater than 40 mbar, for 60 s. The sample prepared in this way was placed in a scanning electron microscope chamber and the measurement was performed. Using the TM-1000 software (Ramsay, New Jersey, US, 2018), the results were recorded on a computer that cooperated with the spectrophotometer.

The flammability of vulcanizates was determined by the oxygen index (*OI*) method. Vulcanizate samples of 50 × 10 × 4 mm were placed vertically in the holder and covered with a quartz column. The construction of the apparatus is protected by the Polish Patent [31]. Inside the column, the sample was perfused with a composition of oxygen (O_2_) and nitrogen (N_2_). The gas flow rate was determined by rotameters. Nitrogen flow was constant and reached 400 L/h, while the oxygen flow was variable and selected to determine the lowest oxygen concentration in the gas composition at which the sample burned during 180 ± 15 s. Samples were ignited for 5 s with a gas burner. After the removal of the fire source, the time of their combustion was measured. The test was performed according to PN-ISO 4589-2. The oxygen index (*OI*) was calculated from Formula (8):

(8) $$OI=\frac{O_{2}}{O_{2}+N_{2}}\times 100\%$$

where *O*_2_ is the oxygen flow rate (L/h) and N_2_ is the nitrogen flow rate (L/h).

The time of burning in the air (*t*_b_) was determined using the same samples as for the oxygen index. The vertically positioned samples were ignited for 5 s using a gas burner. After this time, the burning time of the sample or the time after which the sample was extinguished was measured. The measurement was repeated five times.

## 3. Results and Discussion

The presented work is a continuation of previous research published [30]. Knowing the influence of the amount of copper(I) oxide and copper(II) oxide on the cross-linking of chloroprene rubber and the properties of vulcanizates, fillers were incorporated. This stage aimed to improve the strength properties of vulcanizates and reduce the cost of the composition. The fillers were incorporated into CR compositions containing 3 phr of Cu_2_O or 3 phr of CuO. The applied fillers were silica, carbon black, chalk, kaolin (in the amount of 30 phr each), and MMT (nanofiller, which is montmorillonite modified by ammonium salt-containing hydroxyl groups, in the amount of 5 phr).

### 3.1. Morphology of Cross-Linking Agents and Fillers

**Figure 1:**
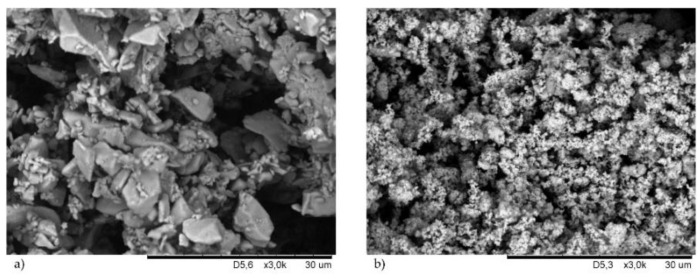
Morphology of cross-linking agents: Cu_2_O (**a**); CuO (**b**).

SEM images of copper(I) oxide and copper(II) oxide are shown in Figure 1. A clear difference in the morphology of both cross-linking substances is visible. The grains of copper(I) oxide of different sizes are shown in Figure 1a. In this case, we noticed that most of the particles were large, with empty areas between them. The structure of copper(II) oxide was different and much more homogeneous (Figure 1b). The CuO particles were small and had a similar size.

**Figure 2:**
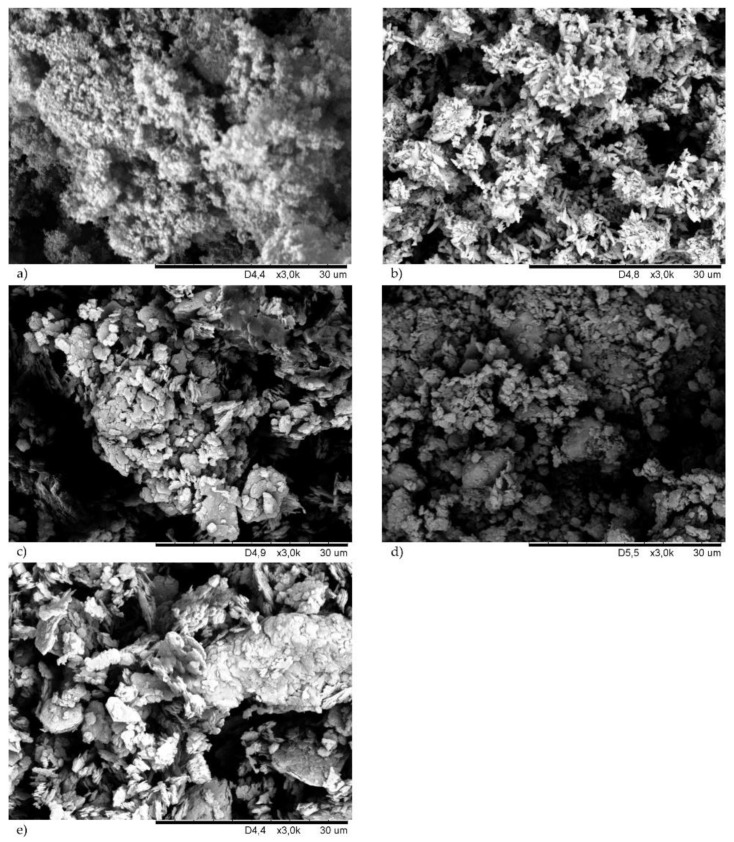
Morphology of fillers: silica (**a**); chalk (**b**); kaolin (**c**); montmorillonite (**d**), carbon black (**e**).

To explain how the physical nature of fillers affects the properties of chloroprene rubber, SEM images of the fillers used (Figure 2) were made. Figure 2a shows the SEM of silica where small spherical particles forming aggregates and agglomerates are visible. Much larger grains, with oblong shapes, belonged to the chalk (Figure 2b). The chalk particles aggregated smaller than silica, but there were more empty areas. In Figure 2c a large single kaolin agglomerate and a lot of voids were observed. The kaolin particles were in the form of larger plates. On the contrary, montmorillonite was characterized by fine spherical grains (Figure 2d). Whereas, carbon black particles were shaped like plates, which in many places formed parallel packages. Moreover, the carbon black grains showed a high tendency to aggregate and agglomerate, and one of them is visible in Figure 2e.

### 3.2. Vulcametric Parameters of Filled CR Compositions

**Figure 3:**
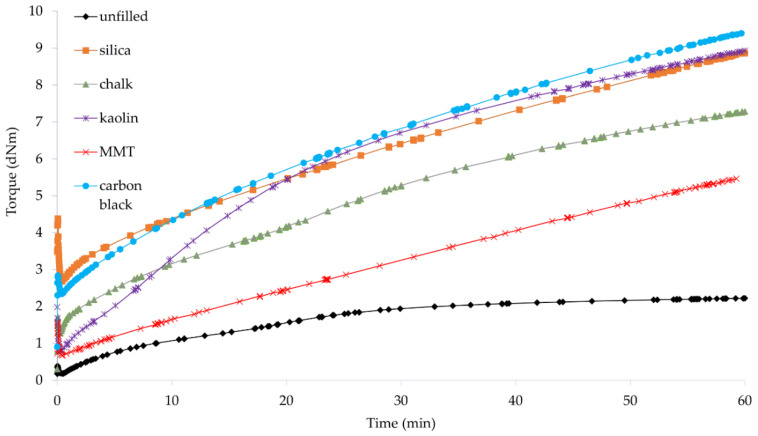
Vulcametric kinetics of filled CR cross-linked with copper(I) oxide (3 phr of Cu_2_O).

**Figure 4:**
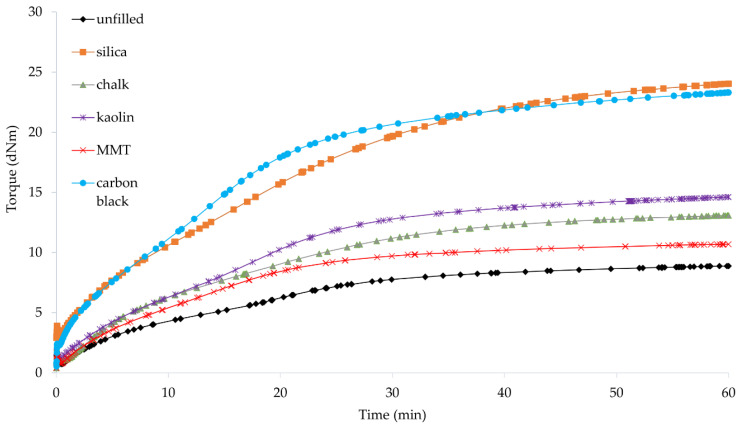
Vulcametric kinetics of filled CR cross-linked with copper(II) oxide (3 phr of CuO).

Rheological methods give information on the entire system as determined by changes in viscoelastic properties or thermodynamic properties. These methods depend not only on the chemical reactions but also on the association, crystallization, and orientation processes that may occur during the preparation of elastomer compositions. Therefore, the determination of vulcametric parameters of the rubber mixture is very important. We have found that the incorporation of fillers shortened the scorch time from 3.1 min (for compositions containing Cu_2_O or CuO) to a value in the range from 0.6 min (for the composition containing Cu_2_O and chalk) to 1.7 min (for the composition containing CuO and chalk) (Table 2, Figure 3 and Figure 4). The exception is the sample containing Cu_2_O and MMT, for which *t*_02_ was equal to 2.6 min. It can be seen that the type of copper oxide used for CR cross-linking had a significant influence on the scorch time. The incorporation of CuO as a cross-linking agent resulted in a reduction of the *t*_02_ parameter (except for the chalk-filled CR).

The vulcanization time did not change significantly as a result of the addition of fillers to the compositions. In the case of compounds containing Cu_2_O, the unfilled sample was characterized by *t*_90_ = 51 min. The incorporation of fillers resulted in a slight reduction (up to 47.6 min in the case of using kaolin) or a slight extension (up to 53 min in the case of using MMT) in the vulcanization time. The only noticeable change can be observed for the composition containing chalk, for which *t*_90_ was 58.8 min. In the case of CR mixtures containing CuO, the vulcanization time was shortened after the fillers were incorporated. The sample without filler had *t*_90_ = 44.9 min. However, the incorporation of the filler resulted in a reduction in the vulcanization time to values ranging from 39.7 min (with the incorporation of silica) to 29.9 min (with the incorporation of MMT). The main factor influencing the vulcanization time, as in the case of the scorch time, was the type of copper oxide. The presence of CuO significantly shortened the *t*_90_ parameter (in the case of CR filled with MMT even by 44%). This is most likely due to the morphology of this copper oxide. As shown in Figure 1b, the structure of copper(II) oxide was much more homogeneous, and its particles were small and of similar size, resulting in a faster cross-linking process, whereas copper(I) oxide was characterized by large grains with empty areas between them, likely extending CR cross-linking.

The incorporation of the fillers into the CR compositions resulted in an increase in the minimum torque. For the unfilled samples, the *M*_min_ value was equal to 0.6 dN · m (for the composition with Cu_2_O or CuO). For samples with a filler, the value of the minimum torque ranged from 0.67 dN · m (for the composition containing Cu_2_O and MMT) to 3.09 dN · m (for the composition containing CuO and silica). The same situation occurred with the torque increment after 45 min of heating—there was an increase in the value as a result of filler incorporation. It is known that the torque increases as the viscosity increases which is typically a result of an increase in filler loading. The viscosity of the filled rubber mixture is dependent on the structure and concentration of the filler, its shape, size, and interaction with the elastomer matrix. A much greater minimum torque was observed after the incorporation of silica or carbon black into the CR mixture, which resulted from the characteristics of these fillers, especially their high agglomeration tendency and their high specific surface area (Figure 2a,e).

The torque measurement also provides data on the effect of fillers on the curing rates of the reactive systems [6]. For unfilled samples, the ∆*M_45_* value was equal to 2.10 and 2.78 dN · m (for compositions with CuO and Cu_2_O, respectively). In general, the increase in the torque increment was greater for compositions containing CuO than for compositions containing Cu_2_O. These results confirm our earlier observations that copper(II) oxide is a more effective cross-linking agent, which is largely related to its homogeneous structure (Figure 1b). In the case of using Cu_2_O, the lowest value was obtained with the incorporation of MMT (∆*M_45_* = 3.75 dN · m), and the highest value with the incorporation of kaolin (∆*M_45_* = 7.11 dN · m). However, in the case of using CuO, the lowest value was obtained with the incorporation of MMT (∆*M_45_* = 9.57 dN · m), and the highest value with the incorporation of carbon black (∆*M_45_* = 19.98 dN · m). In the case of compounds containing copper(II) oxide, the highest values were obtained with the incorporation of active fillers—silica or carbon black. The morphology of both fillers was of great importance for the obtained results. In the case of silica, we observed aggregates, but on their surface, small spherical particles were visible, enabling interactions with CR. The carbon black particles were shaped like plates forming parallel packages into which chains of CR macromolecules and grains of cross-linking agents could easily penetrate, leading to significant degrees of the cross-linking of such compositions. However, ∆*M_45_* values were not dependent on the filler activity, which may indicate a strong inhibitory effect of Cu_2_O on elastomer–filler interactions. The obtained results indicated that the cross-linking speed determined by the *CRI* coefficient was higher if CR was cured with copper(II) oxide. In the case of the CR/CuO/MMT vulcanizate, the speed of vulcanization was the highest (*CRI* = 3.47 min^–1^), and for the CR/Cu_2_O/MMT compound the cure rate index was equal only to 1.98 min^–1^. 

### 3.3. Equilibrium Swelling of Filled CR Vulcanizates

The equilibrium swelling in the study of tested CR vulcanizates showed that the incorporation of fillers changed the equilibrium volume swelling (*Q_V_*) and the equilibrium weight swelling (*Q_w_*), and, thus, the degree of cross-linking. The samples without the filler had a *Q_V_*^T^ value equal to 10.38 cm^3^/cm^3^ and 4.70 cm^3^/cm^3^ (for vulcanizates containing Cu_2_O and CuO, respectively). When Cu_2_O was used for CR cross-linking, the equilibrium swelling values in toluene were dependent on the type of filler used. When MMT or carbon black was incorporated, the *Q_V_*^T^ value (10.68 cm^3^/cm^3^ and 10.92 cm^3^/cm^3^, respectively) did not differ significantly from the value of the unfilled sample. When kaolin was incorporated, the value of the equilibrium volume swelling in toluene increased (*Q_V_*^T^ = 15.89 cm^3^/cm^3^), whereas with the incorporation of chalk and silica, the *Q_V_*^T^ value decreased (6.05 cm^3^/cm^3^ and 6.99 cm^3^/cm^3^, respectively) compared to the sample without a filler. When CuO was used, comparable *Q_V_*^T^ values were observed for the unfilled sample and samples filled with kaolin, MMT, or chalk (4.43 cm^3^/cm^3^, 4.85 cm^3^/cm^3^, and 4.89 cm^3^/cm^3^, respectively). However, with the incorporation of carbon black and silica, the *Q_V_*^T^ value decreased (3.34 cm^3^/cm^3^ and 3.87 cm^3^/cm^3^, respectively) in relation to the unfilled sample (Table 3). These results confirm our previous conclusions regarding these two fillers, which had the greatest impact on reducing the *Q_V_*^T^ value for CR cross-linked with CuO. 

However, in the case of the use of Cu_2_O as a cross-linking agent, the incorporation of silica reduced the *Q_V_*^T^ value (although not to the greatest extent), while the presence of carbon black increased the *Q_V_*^T^ value. 

For the measurements of the equilibrium volume swelling in heptane, when Cu_2_O was used as a cross-linking agent, the *Q_V_*^H^ values of the filled samples were comparable to the result obtained for the unfilled sample (for which *Q_V_*^H^ = 0.40 cm^3^/cm^3^). They ranged from 0.29 cm^3^/cm^3^ (for the vulcanizate containing chalk) to 0.48 cm^3^/cm^3^ (for the vulcanizate containing kaolin). When CuO was used, the *Q_V_*^H^ values of the filled vulcanizates were greater than that of the unfilled sample (*Q_V_^H^* = 0.45 cm^3^/cm^3^). For the sample containing MMT, *Q_V_*^H^ was equal to 0.54 cm^3^/cm^3^, while for the samples containing the remaining fillers, the *Q_V_*^H^ value was 1 cm^3^/cm^3^. 

The influence of fillers on the cross-linking degree was noticeable for vulcanizates containing copper(II) oxide. The incorporation of fillers, especially silica or carbon black, decreased the *Q_V_*^T^ value compared to the unfilled sample. In the case of CR cross-linked with copper(I) oxide, a similar lack of dependence was observed as in the case of vulcametric parameters. This may indicate that in the case of the chloroprene rubber compositions tested, the type of curing agent is of greater importance for the progress of cross-linking than the type of filler used. The presented results confirm the earlier observation that the use of copper(II) oxide leads to a greater degree of cross-linking of chloroprene rubber than the use of copper(I) oxide. This dependence can be easily observed regardless of the type of filler used. This is unambiguously confirmed by the calculated cross-linking degree (*α*_c_), which was much greater for the CR/CuO compositions. For example, the α_c_ value for the CR cured with CuO and filled with carbon black was 0.30, and this parameter for the CR cured with Cu_2_O and filled with carbon black was only 0.09.

### 3.4. Mechanical and Dynamical Properties of Filled CR Vulcanizates

**Figure 5:**
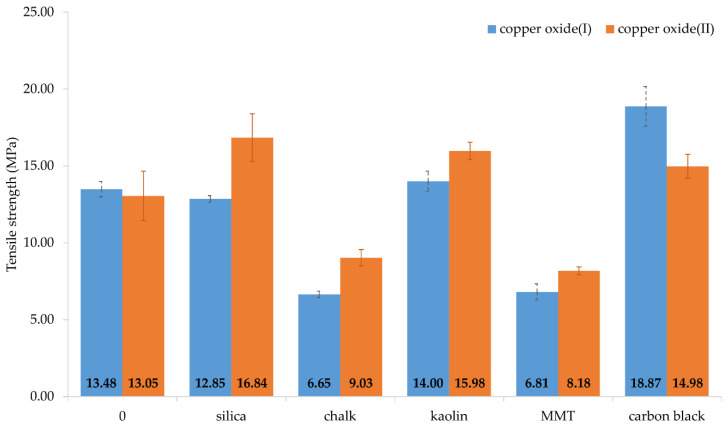
Tensile strength of filled CR vulcanizates; *T* = 160 °C, *t* = 45 min; where: 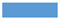 Cu_2_O as a cross-linking agent, 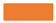 CuO as a cross-linking agent.

Tensile strength testing is by far the most popular method of evaluating filled materials [32,33,34,35,36]. The incorporation of fillers changes the mechanical properties of the prepared vulcanizates. The following factors contribute to the improvement in tensile strength: particle size, particle shape, interaction with the matrix, concentration, and the proper choice of the filler-matrix pair [6]. The tensile strength of the vulcanizates containing Cu_2_O was significantly reduced with the incorporation of chalk and MMT (Table 4, Figure 5). For the samples with these fillers, the *TS*_b_ value was equal to 6.65 and 6.81 MPa, respectively, for chalk- and MMT-containing vulcanizates, while for the unfilled sample it was *TS*_b_ = 13.5 MPa [30]. The vulcanizate containing silica was characterized by comparable strength to that for the unfilled vulcanizate (*TS*_b_ = 12.85 MPa), whereas the presence of kaolin or carbon black resulted in an increase in tensile strength (14.00 MPa and 18.87 MPa, respectively). A similar relationship can be observed in the case of CR cross-linked with CuO. The unfilled sample had a *TS*_b_ value of 13.0 MPa [30]. The incorporation of chalk and MMT decreased *TS*_b_ values to 9.03 MPa and 8.18 MPa, respectively. The presence of silica, kaolin, or carbon black increased the tensile strength to the values of 16.84 MPa, 15.98 MPa, and 14.98 MPa, respectively. 

The elongation at the break is usually inversely proportional to tensile strength which means that increasing the tensile strength of the filled material usually contributes to a decrease in elongation. In most cases, a reduction in elongation is an expected result of vulcanization reinforcement. However, there may also be a situation in which both tensile strength and elongation are increased when fillers are added. Such properties can be obtained in the presence of minor interactions between particles which contribute to a physical cross-linking of a relatively weak matrix. In our research, the elongation at the break for vulcanizates containing Cu_2_O was reduced due to the presence of fillers. For the unfilled sample, *E*_b_ was equal to 752% [30]. The incorporation of chalk, kaolin, or MMT resulted in a slight reduction in *E*_b_ to values from 708–713%. However, in the case of using silica or carbon black, the *E*_b_ values were from 483–484%. In the case of CR cross-linked with CuO, for an unfilled sample, *E*_b_ = 962% [30]. The presence of kaolin or chalk resulted in an *E*_b_ increase in the values from 1123–1125%, and even 1290% in the case of using MMT. The incorporation of silica resulted in a slight reduction in the elongation at the break (*E*_b_ = 869%). In the case of the vulcanizates filled with carbon black, the reduction in the *E*_b_ value was much greater (445%).

The study of the hysteresis allows for determining the amount of mechanical energy used to deform the sample, which is not accumulated in the material and used to return this material to its original form after removing the stress, but instead turns into thermal energy, whereas the calculated Mullins effect is related to the reduction of stress during the same successive deformations of the filled vulcanizates. This effect occurs when bonds are formed between the rubber and filler, but also when agglomerated structures of filler particles are broken. For vulcanizates containing Cu_2_O, the lowest value of the hysteresis (difference of the work between load and unload of the sample during the first cycle, ∆*W*_1_) was obtained for the sample with MMT, for which ∆*W*_1_ = 60 N **·** mm. A comparatively low value (∆*W*_1_ = 71 N **·** mm) was achieved for the vulcanizate filled with chalk (Table 4), whereas the highest *∆W*_1_ values were obtained for samples containing silica or carbon black (163 N **·** mm and 197 N **·** mm, respectively). A similar situation occurred in the case of vulcanizates containing CuO, the lowest value of the hysteresis (∆*W*_1_ = 54 N **·** mm) was obtained for the sample filled with MMT and the highest was for the samples filled with silica or carbon black (265 N **·** mm and 295 N **·** mm, respectively). The lowest value of the Mullins effect for vulcanizates containing Cu_2_O was obtained for the sample filled with chalk (*M*_E_ = 25.9%) and the highest *M*_E_ value was for the sample filled with silica (*M*_E_ = 58.7%). For vulcanizates containing CuO, lower Mullins effect values were achieved for samples filled with MMT or chalk (13.8% and 19.6%, respectively), while the higher *M*_E_ values were 32.2% for the sample filled with kaolin, 37.0% for the sample filled with carbon black, and 39.9% for the sample filled with silica. Higher values of the Mullins effect for vulcanizates containing Cu_2_O may indicate a significant agglomeration of fillers.

To determine the dynamic properties of vulcanizates, the storage modulus (*G′*_max_) and the loss modulus (*G″*_max_) are determined. The *G′*_max_ modulus is a measure of the elastic properties of cured rubber, the so-called immediate (ideal) elasticity. The *G″*_max_ modulus is a measure of the viscous properties of cured rubber and determines its ability to dissipate energy and convert it into heat. The filler type and the cross-linking progress affect the dynamic properties of vulcanizates. The greater the cross-linking progress, the greater the *G′*_max_ modulus, but the lower the *G″*_max_ modulus. In the tested CR vulcanizates, the highest G′_max_ values were achieved for the CR filled with silica (0.862 MPa or 0.530 MPa for the CR cured with Cu_2_O or CuO, respectively) or carbon black (0.882 MPa or 0.549 MPa for the CR cured with CuO or Cu_2_O, respectively). Such high storage modulus values also resulted from the reinforcing nature of the silica and carbon black, as the more active the filler, the greater the *G′*_max_ modulus and *G″*_max_ modulus. However, the loss modulus is a function of the filler’s interface between phases. Therefore, increasing the distance between the filler aggregates (obtained by better mixing, reducing the interactions between the filler particles) or the binding of the filler particles to the rubber reduced the *G″*_max_.

**Figure 6:**
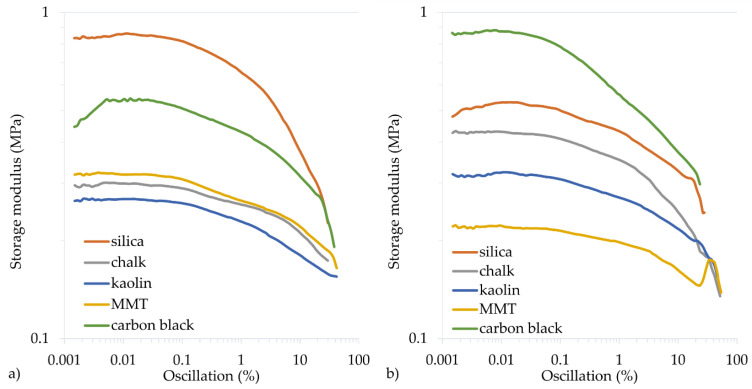
Storage modulus of filled CR cross-linked with copper(I) oxide (**a**) or copper(II) oxide (**b**); *T* = 160 °C, *t* = 45 min.

**Figure 7:**
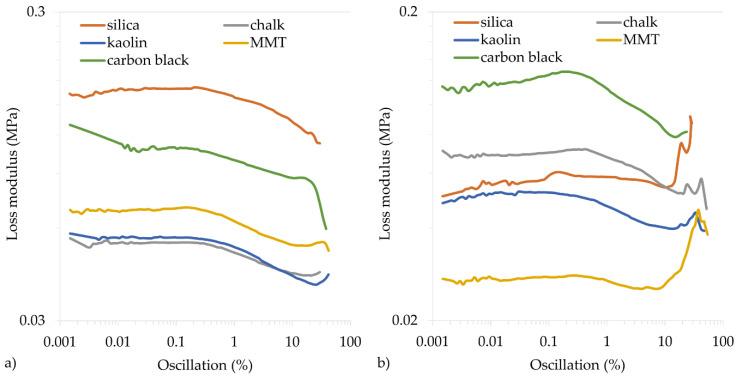
Loss modulus of filled CR cross-linked with copper(I) oxide (**a**) or copper(II) oxide (**b**); *T* = 160 °C, *t* = 45 min.

The calculated Payne effect allows for determining the filler dispersion by undergoing cracking deformation and restoring weak physical bonds connecting the filler agglomerates. For vulcanizates containing Cu_2_O, the Payne effect (∆*G′*) values were the highest for the samples with active fillers, 0.358 MPa for the sample filled with carbon black and 0.636 MPa for the sample filled with silica (Table 5, Figure 6 and Figure 7). In the case of vulcanizates containing CuO, the sample filled with carbon black was characterized by the highest value of the Payne effect (∆*G′* = 0.585 MPa). High values were also obtained for samples filled with silica (∆*G′* = 0.287 MPa) or with chalk (∆*G′* = 0.298 MPa). These studies confirm that the greatest interactions arise between CR and active fillers, which resulted in good mechanical properties and a high cross-linking degree.

The differences between the behavior of carbon black and silica in the CR matrix during the dynamic measurements can be explained by their different energy surface properties. In the case of carbon black, the interactions with the rubber are usually stronger than those in the case of silica, because of the higher dispersion component of the carbon black. On the other hand, silica particles show a stronger tendency to interact in the CR matrix, as well as a greater tendency to agglomerate, which results from the high value of the specific interaction parameter. Thus, the slope of the stress–strain curve in dynamic measurements is related to the type and strength of the filler–rubber and filler–filler interactions.

The analysis of the mechanical and dynamical property measurements of the filled chloroprene rubber vulcanizates shows that the active fillers, i.e., silica and carbon black, create the most developed interactions with the elastomer. Such developed elastomer-filler interactions result in an increase in tensile strength, a reduction in elongation at the break, and an increase in the Payne effect. However, less active fillers also improve the mechanical properties. In the presented systems, the influence of kaolin on the increase in parameters is noticeable, which may indicate strong CR–kaolin interactions, despite the lower development of the specific surface.

### 3.5. Morphology of Filled CR Vulcanizates

**Figure 8:**
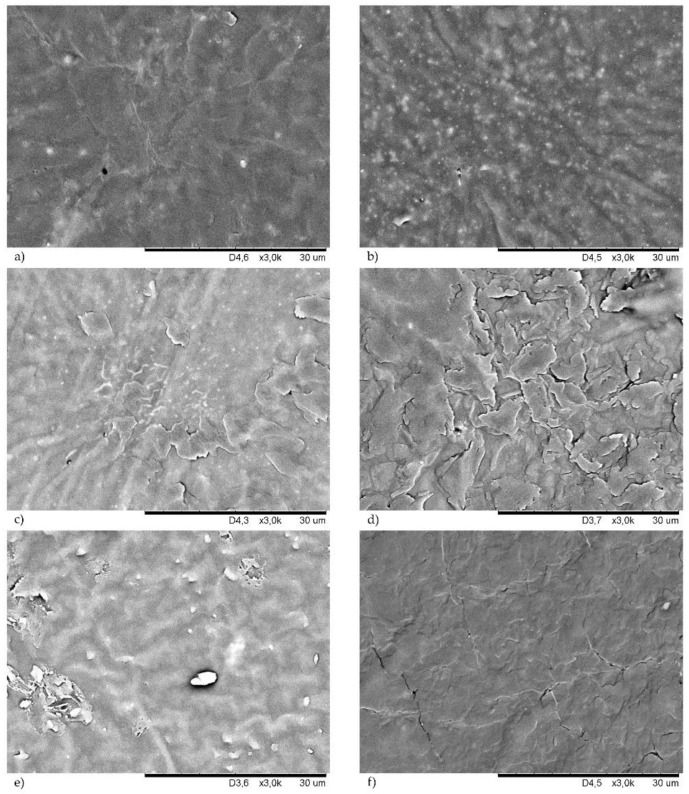
SEM images of the vulcanizates surface of: unfilled CR/Cu_2_O (**a**); CR/Cu_2_O/Si (**b**); CR/Cu_2_O/Ch (**c**); CR/Cu_2_O/Ka (**d**); CR/Cu_2_O/MMT (**e**); CR/Cu_2_O/CB (**f**).

**Figure 9:**
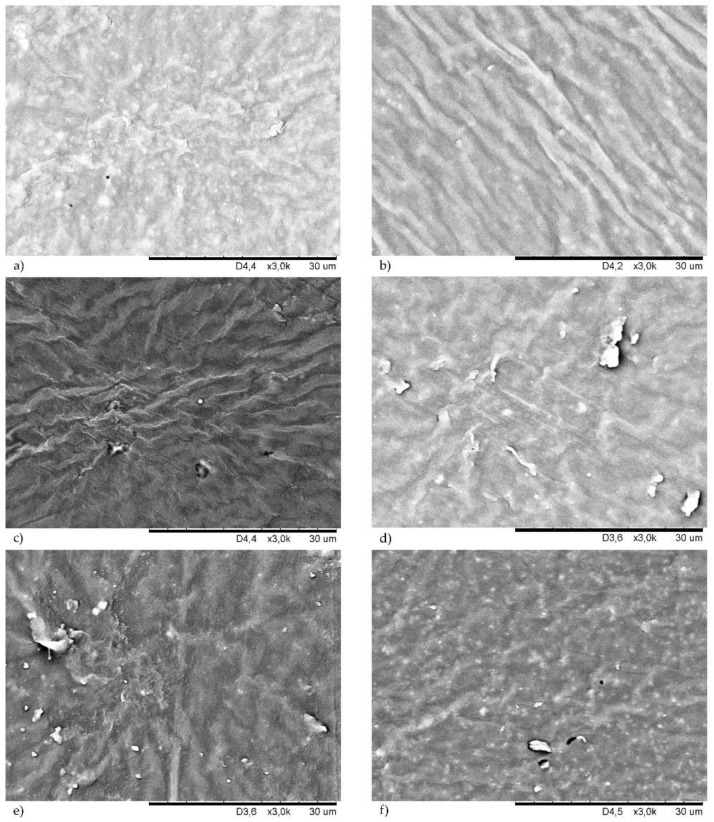
SEM images of the vulcanizates surface of: unfilled CR/CuO (**a**); CR/CuO/Si (**b**); CR/CuO/Ch (**c**); CR/CuO/Ka (**d**); CR/CuO/MMT (**e**); CR/CuO/CB (**f**).

Filler dispersion is an important part of rubber processing technology [12,37,38]. The good dispersion of a filler ensures the good mechanical properties of the final products. The surface morphologies of the studied CR compounds are shown in Figure 8 (samples cured with copper(I) oxide) and in Figure 9 (samples cured with copper(II) oxide). For better comparison, the SEM images of unfilled CR cured with Cu_2_O (Figure 8a) or CuO (Figure 9a) are attached. 

Both morphologies of the unfilled chloroprene rubber were homogeneous, which proved a good dispersion of copper oxides in the CR matrix. The incorporation of the filler changed the surface of the resulting compounds. The presence of silica in the CR cross-linked with Cu_2_O resulted in a rough surface with numerous grooves (Figure 8b). This phenomenon is even more visible in the case of the CR/CuO/Si vulcanizate (Figure 9b). However, it seemed that silica was well-dispersed in the elastomer matrix because no agglomerates or aggregates were observed. The proper dispersion of silica in the CR matrix was likely observed because silica contains large amounts of Si-OH groups on the surface, and, thus, it is considered a highly polar and reactive filler. Silica is, therefore, very compatible with polar chloroprene rubber, giving rise to a good elastomer–filler interaction. The correct dispersion of the silica filler was one of the major factors in the high tensile strength of this vulcanizate. We have observed a similar dependence after the use of carbon black as the CR filler, regardless of the type of copper oxide used. In this case, a good dispersion of the carbon black in the elastomer matrix was also observed (Figure 8f and Figure 9f). Figure 8c and Figure 9c show SEM images of samples filled with chalk, which was evenly dispersed in the CR matrix. In the case of the vulcanizates filled with montmorillonite, better dispersion of this filler was observed for the CR cured with copper(II) oxide (Figure 9e), whereas, the CR/Cu_2_O/MMT compound had numerous aggregates with high brightness on its surface (Figure 8e). 

In most of the vulcanizates tested, there were no differences in the morphology of the samples cross-linked with CuO or Cu_2_O. Vulcanizates filled with kaolin were the only exception here. SEM images of the CR cross-linked with CuO and filled with kaolin showed a few aggregates of this filler (Figure 9d). However, the morphology of the CR cross-linked with Cu_2_O and filled with kaolin was much more diverse (Figure 8d). Stacks of kaolin were marked in this case, and they formed aggregates closer in shape to spherical particles. The presence of agglomerates close to the surface caused surface roughening.

### 3.6. Flammability of Filled CR Vulcanizates

This chapter contains information on the flammability and fire resistance of filled materials because fillers play an important role in limiting the flammability of materials and in reducing the damage and injuries caused by fires. Therefore, the flammability of filled and cross-linked CR compositions was tested. Conventional CR vulcanizates (e.g., CR cured with 5 phr of zinc oxide and 4 phr of magnesium oxide) are characterized by flammability, determined by the oxygen index (*OI*) method. The *OI* value of conventional CR vulcanizates is equal to 26% [39].

However, the compositions obtained in this work were non-combustible materials. The unfilled CR compositions had an *OI* value of 37.3 and 37.5% (respectively, for samples containing Cu_2_O and CuO). This demonstrates the strong flame resistance of the resulting composition, which does not require the incorporation of flame retardants. The use of fillers influenced the change in the oxygen index value. The incorporation of the tested fillers increased the flammability of the vulcanizates to a value of *OI* > 37.5% (Table 6).

Burned samples of rubber materials behave differently, depending on their ingredients. All vulcanizates burned with a blue glow of smoking flame, which was the result of using copper oxides. The amount of soot emitted during combustion was dependent on the composition tested. Samples containing carbon black were characterized by the emission of large fragments of soot in significant amounts. For the chalk-containing, MMT-containing, and unfilled samples a medium amount of soot was emitted. In turn, the samples containing silica and kaolin were characterized by a low amount of soot. The cross-linked CR compositions containing silica, kaolin, carbon black, but also samples with Cu_2_O and MMT, did not burn completely during the measurements. This means that after the measurement there were fragments of the sample that were not covered and consumed by the flame. After the measurement, there were no residues of the chalk-containing samples left—they were completely burned. The sample of the CR vulcanizate containing CuO and MMT retained shape after measurement. The unfilled CR composition samples also retained shape after burning but were brittle and easy to break.

## 4. Conclusions

In summary, chloroprene rubber cross-linked with copper(I) oxide or copper(II) oxide can be filled with both reinforcing, semi-reinforcing, and non-reinforcing fillers. The type of filler had a large impact on the degree of cross-linking of chloroprene rubber and mechanical, dynamical, and structural properties of vulcanizates. The second parameter of great importance for the above-mentioned properties was the type of copper oxide used as the cross-linking substance. The degree and speed of cross-linking of the filled CR were higher when the CR was cured with copper(II) oxide. Among the fillers used, the presence of carbon black or silica ensured the highest degree of CR cross-linking and the best strength properties. Surprisingly, high tensile strength was also obtained in the case of CR cross-linked with copper(II) oxide and filled with kaolin. The Payne effect was greatest for vulcanizates filled with silica or carbon black, which confirmed the greatest interactions between CR and active fillers, which resulted in good mechanical properties and a high degree of cross-linking. These good filler–elastomer interactions also determined the proper dispersion of silica and carbon black in the CR matrix. It was noticed that the CR cross-linked with copper(II) oxide was characterized by a slightly better dispersion of the components in comparison to the CR cross-linked with copper(I) oxide. Moreover, it was found that the use of copper(II) oxide as the CR curing agent, regardless of the filler used, led to a much lower Mullins effect, which indicated better dispersion of the fillers in the matrix of such cross-linked chloroprene rubber. The flammability tests indicated that all produced vulcanizates were characterized by a high oxygen index, which allows them to be classified as non-flammable materials.

## Figures and Tables

**Table 1:** Formulation of filled chloroprene rubber (CR) composites and their designations.

Component Content (phr)										
CR	100	100	100	100	100	100	100	100	100	100
SA	1	1	1	1	1	1	1	1	1	1
Cu_2_O	3	-	3	-	3	-	3	-	3	-
CuO	-	3	-	3	-	3	-	3	-	3
Silica	30	30	-	-	-	-	-	-	-	-
Chalk	-	-	30	30	-	-	-	-	-	-
Kaolin	-	-	-	-	30	30	-	-	-	-
MMT	-	-	-	-	-	-	5	5	-	-
CB	-	-	-	-	-	-	-	-	30	30
Symbol	Cu_2_O/Si	CuO/Si	Cu_2_O/Ch	CuO/Ch	Cu_2_O/Ka	CuO/Ka	Cu_2_O/MMT	CuO/MMT	Cu_2_O/CB	CuO/CB

**Table 2:** Vulcametric parameters of the filled CR compositions determined at the temperature of 160 °C.

Symbol	*t*_02_ (min)	*t*_90_ (min)	*M*_min_ (dN · m)	Δ*M_45_* (dN · m)	*CRI* (min^−1^)
Cu_2_O/Si	1.3	51.8	2.68	4.96	1.98
CuO/Si	1.1	39.7	3.09	19.71	2.59
Cu_2_O/Ch	0.6	58.8	1.27	5.11	1.72
CuO/Ch	1.7	35.6	1.01	11.57	2.95
Cu_2_O/Ka	1.5	47.6	0.79	7.11	2.16
CuO/Ka	1.1	34.3	1.09	12.89	3.01
Cu_2_O/MMT	2.6	53.0	0.67	3.75	1.98
CuO/MMT	1.1	29.9	0.76	9.57	3.47
Cu_2_O/CB	1.5	50.7	2.34	6.04	2.03
CuO/CB	0.8	34.0	2.28	19.98	3.01

*t*_02_—scorch time, *t*_90_—cure time, *M*_min_—minimum torque, Δ*M_45_*—torque increment after 45 min of heating, CRI—cross-linking rate.

**Table 3:** Values of equilibrium swelling of filled CR vulcanizates; *T* = 160 °C, *t* = 45 min.

Symbol	*Q_V_*^T^ (cm^3^/cm^3^)	*Q_V_*^H^ (cm^3^/cm^3^)	*Q_w_*^T^ (mg/mg)	*Q_w_*^H^ (mg/mg)	*α*_c_
Cu_2_O/Si	6.99 ± 0.14	0.44 ± 0.15	4.94 ± 0.10	0.24 ± 0.09	0.14
CuO/Si	3.87 ± 0.07	1.00 ± 0.01	2.74 ± 0.05	0.55 ± 0.01	0.26
Cu_2_O/Ch	6.05 ± 0.07	0.29 ± 0.05	4.28 ± 0.06	0.16 ± 0.03	0.17
CuO/Ch	4.89 ± 0.11	1.00 ± 0.02	3.46 ± 0.08	0.55 ± 0.01	0.20
Cu_2_O/Ka	15.89 ± 0.39	0.48 ± 0.03	11.24 ± 0.28	0.26 ± 0.02	0.06
CuO/Ka	4.43 ± 0.06	1.02 ± 0.02	3.14 ± 0.05	0.56 ± 0.01	0.23
Cu_2_O/MMT	10.68 ± 0.09	0.31 ± 0.04	7.55 ± 0.07	0.17 ± 0.03	0.09
CuO/MMT	4.85 ± 0.11	0.54 ± 0.03	3.43 ± 0.08	0.30 ± 0.02	0.21
Cu_2_O/CB	10.92 ± 0.11	0.38 ± 0.03	7.73 ± 0.08	0.21 ± 0.02	0.09
CuO/CB	3.34 ± 0.04	1.00 ± 0.05	2.37 ± 0.03	0.56 ± 0.03	0.30

*Q_V_*^T^*, Q_V_*^H^—equilibrium volume swelling in toluene or heptane, respectively; *Q_w_*^T^*, Q_w_*^H^—equilibrium weight swelling in toluene or heptane; *α*_c_—cross-linking degree determined in toluene.

**Table 4:** Mechanical properties of filled CR vulcanizates; *T* = 160 °C, *t* = 45 min.

Symbol	*S*_e100_ (MPa)	*S*_e200_ (MPa)	*S*_e300_ (MPa)	*TS*_b_ (MPa)	*E*_b_ (%)	∆*W*_1_ (N · mm)	*M*_E_ (%)
Cu_2_O/Si	3.32 ± 0.06	5.71 ± 0.20	8.54 ± 0.39	12.85 ± 0.22	483 ± 26	163	58.7
CuO/Si	2.02 ± 0.10	3.60 ± 0.07	5.47 ± 0.30	16.84 ± 1.55	869 ± 47	265	39.9
Cu_2_O/Ch	0.65 ± 0.04	0.79 ± 0.04	0.98 ± 0.04	6.65 ± 0.21	708 ± 23	71	25.9
CuO/Ch	1.12 ± 0.06	1.60 ± 0.09	2.05 ± 0.12	9.03 ± 0.53	1125 ± 39	92	19.6
Cu_2_O/Ka	1.33 ± 0.06	2.01 ± 0.07	2.86 ± 0.09	14.00 ± 0.66	710 ± 11	11	38.7
CuO/Ka	1.74 ± 0.01	2.93 ± 0.05	4.05 ± 0.10	15.98 ± 0.56	1123 ± 25	201	32.2
Cu_2_O/MMT	0.73 ± 0.06	0.92 ± 0.09	1.16 ± 0.15	6.81 ± 0.52	713 ± 16	60	29.7
CuO/MMT	0.83 ± 0.01	1.12 ± 0.01	1.36 ± 0.02	8.18 ± 0.26	1290 ± 10	54	13.8
Cu_2_O/CB	1.53 ± 0.23	4.22 ± 0.22	8.53 ± 1.32	18.87 ± 1.29	484 ± 31	197	48.4
CuO/CB	3.14 ± 0.05	6.30 ± 0.08	10.1 ± 0.20	14.98 ± 0.77	445 ± 41	295	37.0

*S*_e100_, *S*_e200_, *S*_e300_—stress at elongation of 100%, 200%, or 300%, respectively, *TS*_b_—tensile strength, *E*_b_—elongation at break, ∆*W*_1_—hysteresis, *M*_E_—Mullins effect.

**Table 5:** Payne effect of filled CR vulcanizates; *T* = 160 °C, *t* = 45 min.

Symbol	∆*G′* (MPa)	*G′*_max_ (MPa)	*G″*_max_ (MPa)
Cu_2_O/Si	0.636	0.862	0.171
CuO/Si	0.287	0.530	0.092
Cu_2_O/Ch	0.127	0.301	0.056
CuO/Ch	0.298	0.433	0.072
Cu_2_O/Ka	0.114	0.269	0.058
CuO/Ka	0.169	0.324	0.052
Cu_2_O/MMT	0.158	0.323	0.070
CuO/MMT	0.084	0.222	0.046
Cu_2_O/CB	0.358	0.549	0.137
CuO/CB	0.585	0.882	0.128

∆*G′*—Payne effect, *G′*_max_—maximum storage modulus, *G″*_max_—maximum loss modulus.

**Table 6:** Flammability of filled CR vulcanizates; *T* = 160 °C, *t* = 45 min.

Symbol	*OI* (%)	*t*_b_ (s)	Observation
Cu_2_O *	37.3	175	The sample burned completely, an orange flame with a blue glow covered the entire sample volume, a smoking flame, a medium amount of soot, after burning the sample retained shape but was brittle, it was easy to break,
CuO *	37.5	187	The sample burned completely, an orange flame with a blue glow covered the entire sample volume, a smoking flame, a medium amount of soot, after burning the sample retained shape but was brittle, it was easy to break.
Cu_2_O/Si	>37.5	23	The sample did not burn completely, an orange flame with a blue glow layer covered the sample, a smoking flame, a small amount of soot.
CuO/Si	>37.5	<5	The sample did not burn completely, an orange flame with a blue glow layer covered the sample, a smoking flame, a small amount of soot.
Cu_2_O/Ch	>37.5	218	The sample burned completely, an orange flame with a blue glow covered the entire sample volume, a smoking flame, a medium amount of soot, sample burned completely, there were no residues after combustion of the sample.
CuO/Ch	>37.5	239	The sample burned completely, an orange flame with a blue glow covered the entire sample volume, a smoking flame, a medium amount of soot, sample burned completely, there were no residues after combustion of the sample.
Cu_2_O/Ka	>37.5	<5	The sample did not burn completely, an orange flame with a blue glow layer covered the sample, a smoking flame, a small amount of soot.
CuO/Ka	>37.5	41	The sample did not burn completely, an orange flame with a blue glow layer covered the sample, a smoking flame, a small amount of soot.
Cu_2_O/MMT	>37.5	204	The sample did not burn completely, an orange flame with a blue glow covered almost the entire sample volume, a smoking flame, a medium amount of soot.
CuO/MMT	>37.5	216	The sample burned completely, an orange flame with a blue glow covered the entire sample volume, a smoking flame, a medium amount of soot, the sample retained shape after burning.
Cu_2_O/CB	>37.5	60	The sample did not burn completely, an orange flame with a blue glow layer covered the sample, a smoking flame, a very large amount of soot.
CuO/CB	>37.5	60	The sample did not burn completely, an orange flame with a blue glow layer covered the sample, a smoking flame, a very large amount of soot.

*—unfilled CR vulcanizates.

## Data Availability

Data sharing not applicable.
